# Parents with periodontitis impact the subgingival colonization of their offspring

**DOI:** 10.1038/s41598-020-80372-4

**Published:** 2021-01-14

**Authors:** Mabelle Freitas Monteiro, Khaled Altabtbaei, Purnima S. Kumar, Márcio Zaffalon Casati, Karina Gonzales Silverio Ruiz, Enilson Antonio Sallum, Francisco Humberto Nociti-Junior, Renato Corrêa Viana Casarin

**Affiliations:** 1 Periodontics Division, Department of Prosthodontics and Periodontics, Piracicaba Dental School, University of Campinas, Piracicaba, SP Brazil; 2 Department of Periodontology, School of Dentistry, University of Alberta, Edmonton, Canada; 3 Department of Periodontology, College of Dentistry, The Ohio State University, Columbus, OH USA

**Keywords:** Dental diseases, Infection, Predictive markers, Bacteria, Clinical microbiology, Microbial communities, High-throughput screening

## Abstract

Early acquisition of a pathogenic microbiota and the presence of dysbiosis in childhood is associated with susceptibility to and the familial aggregation of periodontitis. This longitudinal interventional case–control study aimed to evaluate the impact of parental periodontal disease on the acquisition of oral pathogens in their offspring. Subgingival plaque and clinical periodontal metrics were collected from 18 parents with a history of generalized aggressive periodontitis and their children (6–12 years of age), and 18 periodontally healthy parents and their parents at baseline and following professional oral prophylaxis. 16S rRNA amplicon sequencing revealed that parents were the primary source of the child's microbiome, affecting their microbial acquisition and diversity. Children of periodontitis parents were preferentially colonized by *Filifactor alocis*, *Porphyromonas gingivalis, Aggregatibacter actinomycetemcomitans*, *Streptococcus parasanguinis*, *Fusobacterium nucleatum* and several species belonging to the genus *Selenomonas* even in the absence of periodontitis, and these species controlled inter-bacterial interactions. These pathogens also emerged as robust discriminators of the microbial signatures of children of parents with periodontitis. Plaque control did not modulate this pathogenic pattern, attesting to the microbiome's resistance to change once it has been established. This study highlights the critical role played by parental disease in microbial colonization patterns in their offspring and the early acquisition of periodontitis-related species and underscores the need for greater surveillance and preventive measures in families of periodontitis patients.

## Introduction

Periodontitis is an infection-mediated inflammatory disease, in which the primary etiologic factor is the subgingival biofilm. One phenotype of this disease that has been identified specifically in young, circumpubertal individuals demonstrates a rapid rate of progression, resulting in precocious tooth loss^1^. Studies have reported that this phenotype, known as aggressive periodontitis (and currently classified as grade C periodontitis^2^), presents a familial aggregation. The aggregation of cases in the same family is estimated to be 50%^3,4^, and both vertically transmitted genetic factors (such as those responsible for microbial colonization or host response), as well as shared environmental factors (such as oral hygiene and smoking), can increase the risk of developing periodontitis^4–6^. Thus, elucidating these susceptibility elements and the familial component of this disease is critical to understanding disease pathogenesis.

One of the most plausible causes for the familial aggregation, besides the probable genetic component, is the transmission of microorganisms within family members and early colonization by putative periodontal pathogens^4,7,8^. These events could favor the establishment of a dysbiotic ecosystem, increasing the risk for initial and severe periodontal destruction. Studies in many body sites have demonstrated that the parents' microbiome is a critical determinant of microbial colonization in their offspring^9–11^. It is also known that vertically transmitted dysbiotic gut biomes contribute to obesity in the child^12^. In this context, it is logical that the parent could impact their offspring's oral health by vertical transmission of oral bacteria.

There is substantial evidence that specific periodontal pathogens demonstrate a familial aggregation, and in some instances, there is evidence of vertical transmission from mother to child^13–15^. For instance, maternal oral condition (periodontal disease, hygiene habits, and colonization with specific pathogens) is a risk for periodontal disease in the offspring, and children from diseased mothers are more frequently affected with periodontal disease^13^. Studies have also demonstrated that children of parents suffering from Generalized Aggressive Periodontitis (GAgP) present higher colonization by *A. actinomycetemcomitans*, an established periodontal pathogen, in saliva and subgingivally^14–16^, are at 16 times higher risk to be colonized by that microorganism if the parents are also colonized for it^14^, and present higher levels of clinical and subgingival inflammation^16^. However, literature only points to the acquisition of specific pathogens, and the influence of parental periodontal status on microbial colonization of the child is not known. This study combined a longitudinal interventional study of parent–child dyads with metataxonomics to evaluate the influence of parental periodontal disease on the subgingival microbial community and oral health of their children.

## Results

No differences were evident in the demographic characteristics of both groups (Table 1). 12 mothers and 8 female children were included in each group; the mean ages being 36.50 ± 4.32 years for parents of periodontitis group and 9.70 ± 2.16 for their children and 36.46 ± 3.81 and 9.55 ± 1.97 for the healthy parents and their offspring respectively. Parents with periodontitis demonstrated a higher level of PD, CAL, and BoP when compared to healthy parents (*p* < 0.05, Mann–Whitney test). Children of parents with periodontitis presented higher BoP than children from healthy parents (*p* < 0.05, Mann–Whitney test). Plaque control reduced the PI for both groups and GI and BoP in the periodontitis group at 3 months (*p* < 0.05, Wilcoxon test). At 3 months, no clinical differences were observed between groups (*p* > 0.05, Mann–Whitney test).

**Table 1:** Clinical data on children and their parents.

	Parent	Children	
		Baseline	3 months
**PI (%)**			
Periodontitis	34.2 ± 14.1	55.7 ± 13.9	32.8 ± 17.3^b^
Health	38.5 ± 16.9	50.6 ± 22.1	36.4 ± 14.6^b^
**GI (%)**			
Periodontitis	8.9 ± 2.0	24.8 ± 13.2	17.3 ± 8.0^b^
Health	10.5 ± 2.6	20 ± 12.1	20.6 ± 13.5
**BoP (%)**			
Periodontitis	35.9 ± 11.7	37.3 ± 14.4	33.9 ± 13.6
Health	25.5 ± 12.3^a^	25.8 ± 12.1^a^	25.3 ± 13.9
**PD (mm)**			
Periodontitis	4.3 ± 0.6	–	–
Health	3.0 ± 0.5^a^	–	–
**CAL (mm)**			
Periodontitis	5.1 ± 1.0	–	–
Health	3.0 ± 0.5^a^	–	–

SD, Standard deviation; PI, Plaque Index; GI, Gingival Index; PD, Probing depth; BoP, Bleeding on probing; CAL, Clinical Attachment Leve.

^a^Represents differences between Periodontitis and Health groups (*p* < 0.05, Mann–Whitney test).

^b^Represents differences between Baseline and 3 months for children from each group (*p* < 0.05, Wilcoxon test).

### Disease-naïve children of parents with periodontitis host a pathogen-rich subgingival microbial community

**Figure 1:**
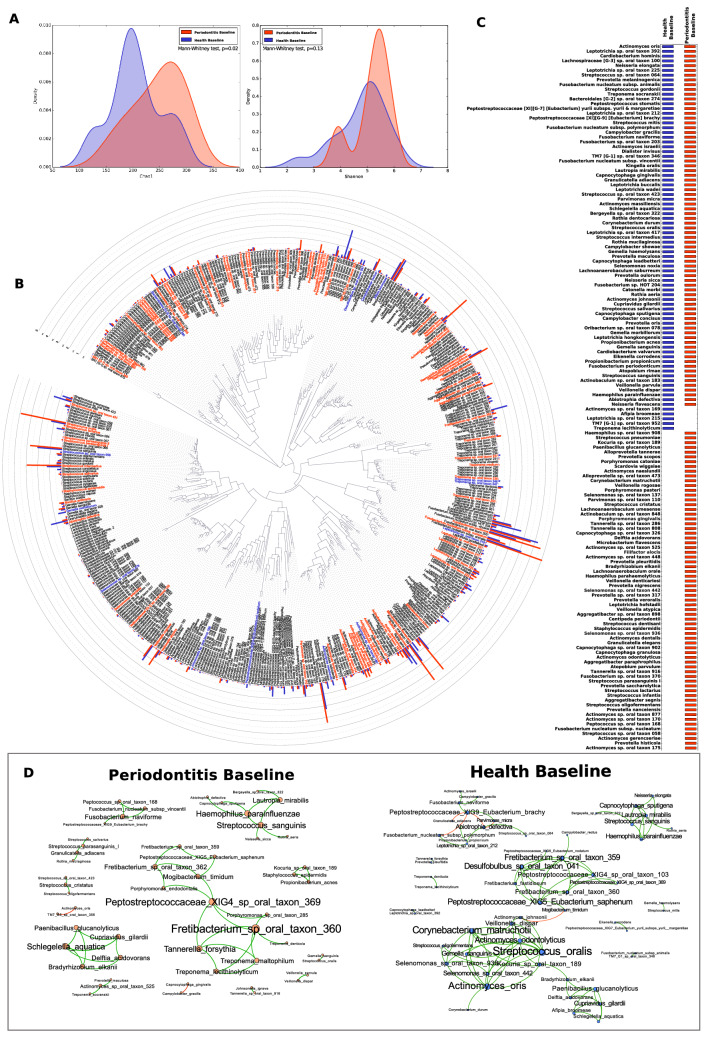
The subgingival microbiome of the offspring of generalized aggressive periodontitis patients and periodontally healthy individuals at baseline. (**A**) α-diversity: Chao1, and Shannon estimators. (**B**) Representation of the phylogenetic tree (iToL), the relative abundance of bacteria and species differentially abundant between groups (*p* < 0.05, FDR-adjusted Wald Test, DESeq2). The external bars represent the relative abundance of each taxon, and the colored names represent species that were significantly more abundant in each group. (**C**) The core microbiome, representing species that were present in ≥ 80% of the subjects in a group. (**D**) Network co-occurrence analysis. The graphs describe the SparCC correlations between the species abundance in periodontitis and health groups (r > 0.75, *p* < 0.01). The green edges represent a positive correlation, while a red edge represents a negative correlation between two nodes. Each node represents one bacterium, and the node size is proportional to the number of correlations.

Figure 1 represents the baseline microbial assemblages in children of both groups. Children from the periodontitis group demonstrated higher species richness (*p* = 0.02, Mann–Whitney test of Chao 1 index) and similar diversity (*p* = 0.13, Mann–Whitney test of Shannon index, Fig. 1A), and higher relative abundances of certain species in the subgingival microbiome (Fig. 1B) when compared to the healthy group. A larger common core of species was observed in children in the periodontitis group, indicating greater microbial homogeneity. Bacteria such as *Filifactor alocis*, *Porphyromonas gingivalis, Streptococcus parasanguinis*, *Fusobacterium nucleatum* subsp*. nucleatum* and several species belonging to the genus *Selenomonas* were exclusively observed in the core of the periodontitis group, suggesting that some periodontitis-related bacteria are highly prevalent in children of diseased parents at an early age (Fig. 1C). Co-occurrence analysis revealed very few inter-bacterial interactions, with approximately 40% of the connections centered on species belonging to the genera *Streptococcus, Fretibacterium, Treponema, Fusobacterium, Tannerella.* In contrast, a more complex network was observed in the healthy group, with five highly connected hubs. Approximately 40% of the connections were driven by species belonging to the genera *Actinomyces*, *Streptococcus*, *Fretibacterium*, *Corynebacterium,* and *Selenomonas*. (Fig. 1D).

### A parent’s microbiome determines microbial acquisition and diversity in their offspring

**Figure 2:**
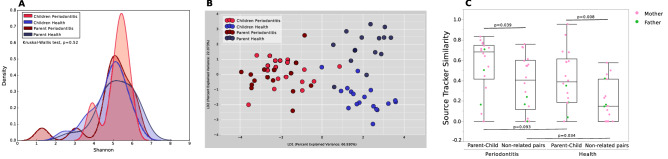
The similarity between parents' and children's microbiome. (**A**) α-diversity, Shannon estimator. (**B**) β-diversity, LDA of Morisita-Horn Dissimilarity Index showing the children and their parents in both groups. (**C**) Source Tracker results with the similarity between a parent and their child and a parent and a non-related child in the periodontitis and health groups.

Parents and children from both groups presented similar alpha diversity (*p* = 0.52, Kruskal–Wallis test of Shannon index, Fig. 2A). On the other hand, parents in healthy and periodontitis groups demonstrated significant difference in beta diversity (*p* = 0.019, Adonis test of LDA of Morisita–Horn dissimilarity index, Fig. 2B) as did their offpsring (*p* = 0.01, Adonis test), while no differences could be identified between parent–child dyads in both periodontitis and healthy groups (*p* = 0.695 and *p* = 0.998 respectively, Adonis test). The similarity between adults and their descendants was greater than that between non-related individuals for periodontitis and healthy groups (Mann–Whitney test of SourceTracker analysis, *p* = 0.03 and *p* = 0.008, respectively, Fig. 2C), highlighting the significant influence of parental periodontal disease in determining the species that colonize their offspring. While parents with periodontitis demonstrated a median of 70% similarity with their offspring, the median similarity was below 40% between healthy parent–child dyads (Mann–Whitney test, *p* = 0.09).

### The microbiome, once established, is resilient to change

**Figure 3:**
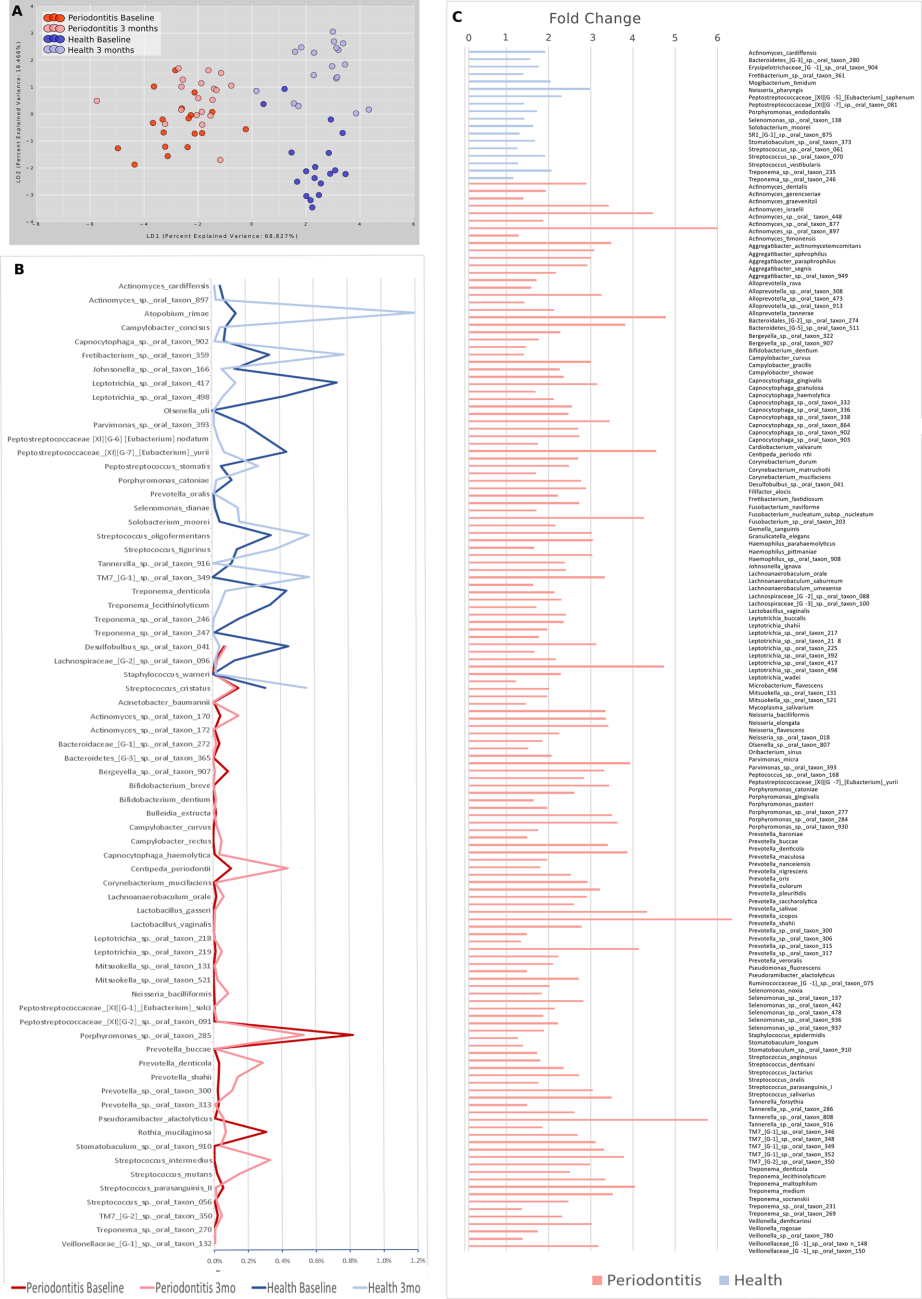
Longitudinal evaluation of the microbiome at baseline and three months after plaque control. (**A**) β-diversity, LDA of Morisita-Horn Dissimilarity Index. (**B**) Change in the relative abundance of species over time (*p* < 0.05, FDR-adjusted Wald Test, DESeq2). (**C**) Species differentially abundant between periodontitis and health groups after plaque control. The length of the bar indicates the fold-difference between groups. The pink bars represent the bacteria more abundant in children from periodontitis parents, the blue bars the bacteria more abundant in children from healthy parents (*p* < 0.05, FDR-adjusted Wald Test, DESeq2).

Although plaque control resulted in similar clinical improvements in both groups, the magnitude of the oral microbiome shift was different between groups (Fig. 3A). LDA of Morisita-Horn dissimilarity index demonstrated the formation of statistically significant clusters, and the periodontitis and healthy groups were separated on the first dimension (*p* < 0.05, Adonis test), while the second dimension separated the microbiomes at baseline and three months. No difference was noted between baseline and 3 months for the periodontitis (*p* = 0.484, Adonis test) or healthy groups (*p* = 0.546, Adonis test), indicating that vertical transmission might be a more significant driver of colonization than environmental factors such as oral hygiene. Longitudinal analysis indicated that oral hygiene resulted in changes in rare taxa in both groups (those accounting for ≤ 0.1% of the overall abundance, Fig. 3B). At 3 months, 162 species were found to be differentially abundant between groups, with 144 species more abundant in the periodontitis group than the healthy group (Fig. 3C), including several higher gram-negative and anaerobic organisms, and periodontal pathobionts such as *Aggregatibacter actinomycetemcomitans, Filifactor alocis, Streptococcus parasanguinis, Fusobacterium nucleatum subsp. nucleatum, Porphyromonas gingivalis, Treponema denticola,* and *Tannerella forsythia.*

## Discussion

The influence of a parent’s microbiome, especially mothers, on the composition of the microbiome of a child is widely described in the literature^7–11^. The influence impacts many body sites, such as skin, gut, and mouth. Moreover, the similarity between environments correlates significantly with the amount of contact between the microbial communities^17^. All of this supports vertical transmission (transmission from parents to their children) of microorganisms as an essential way of acquiring oral microbiota from family members who have close contact and share hygiene, feeding, and social habits.

Alteration of the microbial community in parents, as occurs in some infectious diseases, could be a determinant in promoting the early contact of children with pathogens and the establishment of dysbiotic communities^18^. Indeed, studies from the gut microbiome have demonstrated that mothers who are obese or diabetic, for example, transfer a microbiome characteristic of these diseases to their children^19,20^. Using a longitudinal study design and a familial approach, we confirmed a prominent role for parental periodontitis on the microbiome of the offspring, and we demonstrated resistance to shift in this early acquired microbiome, indicating a high degree of heritability.

Even though none of the children demonstrated attachment or bone loss, those from the periodontitis group presented higher levels of clinical inflammation and worse periodontal status, a result that corroborates previous studies demonstrating that parents’ periodontal status could impact the clinical condition of their offspring^13–16^. The worse clinical condition was accompanied by an alteration in species richness, beta diversity, and core bacterial species. The core microbiome is a collection of species highly prevalent in a cohort, and one that probably plays a central structural and functional role for that community^21^. Established periodontal pathogens such as *F. alocis, S. parasanguinis, F. nucleatum, P.gingivalis,* and *Selenomonas* genera were identified in the periodontitis core, highlighting their importance to that environment. More specifically, *A. actinomycetemcomitans, F. alocis,* and *S. parasanguinis*, organisms that have been identified as a consortium related to risk and future bone loss in adolescents^22^, were found to be more abundant and as part of the core microbiome in children in the periodontitis group. Moreover, these alterations in community composition extended to inter-species networks. In the periodontitis group, species were sparsely connected, and most of the connections were driven by genera such as *Treponema*, *Fusobacterium* and *Tannerella*, usually associated with a mature and disease-associated biofilm^6,23^.

The consequence of subgingival dysbiosis at an early age to periodontal health has not yet been elucidated. However, findings from other body sites, such as the gut, have demonstrated that dysbiosis early in life can negatively impact the maturation of the immune system and the regulation of the host response to microbial aggression^24,25^. The crosstalk between the commensal microbiota and the immune system is essential for training the host response in recognizing the bacteria, in promoting immunological tolerance, and shaping the ideal inflammatory response^26^. Furthermore, any alteration on that homeostasis could change metabolic pathways and promote a non-resolving inflammation, driving the environment for a disease condition^26^. Studies that correlate oral dysbiosis during infancy with altered development of the immune system and allergies^27^ and the evidence that specific microorganisms, such as *P. gingivalis*, could modulate dysbiosis and inflammation in the periodontium^28^ further support this idea.

One of the primary drivers of oral dysbiosis is poor oral hygiene, which in turn is influenced by parental attitudes to home care^29^. Since the offspring of parents with periodontitis demonstrated higher plaque levels than the healthy group, we examined the microbiome following professionally administered prophylaxis and monitored oral hygiene visits. Interestingly, even though children from the periodontitis group improved their clinical condition to such a level that their clinical health was not significantly different from the control group at three months, the impact on the microbial community was limited. Although some species did change over time in this group, the changes were concentrated in less abundant taxa, and not large enough to impact diversity metrics. On the other hand, oral prophylaxis and oral hygiene had a more significant effect on the microbiome of the healthy group, altering a higher number of taxa and impacting their proportions. However, as with the periodontitis group, this shift was not large enough to change the diversity metrics. It suggests that once the microbial community is established, it becomes resilient to shift and impactful events would be necessary to alter that structure. Resilience is a property of a microbial community that determines how fast, and to what extent, it will recover its original taxonomic and functional structure in the aftermath of a perturbation^30,31^. Therefore, events more powerful than supragingival plaque removal would be necessary to initiate profound shifts in the established microbial community. Thus, the early acquisition and stable establishment of a microbiome that is not health-compatible, and one that demonstrates a high degree of resilience can serve to increase the risk for disease later in life.

Although this study points to the early acquisition of a pathogenic microbiome by vertical transmission and its transition to a dysbiotic community as a possible factor associated with familial aggregation of periodontitis and its onset, it is not clear if and what host factors are related to dysbiosis, and what genetics and behavioral factors alter the microbial composition and the host response to the aggression. Evidence from previous studies supports the hypothesis that host genetics also exerts selective pressure on the microbiota^32–34^, and by altering the host response or the inflammatory pattern locally, it could produce the ecologic conditions necessary for the outgrowth of specific pathogens and the dysbiosis occurrence^34^. It is beyond the scope of this study to examine the heritability of host genotypic factors, however, irrespective of the cause of dysbiosis, it is known that a pathogenic subgingival environment since childhood is associated with an increased risk for the development of future periodontal disease^22,35,36^. Hence, the evidence that the parent's periodontal status can affect the periodontal condition of their children should be an essential tool in the clinic, focusing on prevention, early diagnosis, and clinical management of this population. This study demonstrates that the alteration on the subgingival environment occurs at an early age; however, when this microbiome is acquired, the mechanisms by which this dysbiotic microbiome predisposed to disease, and the potential impact of targeted microbial modulation therapies either alone or in conjunction with mechanical biofilm disruption are not known and should be the focus of future studies.

In conclusion, the parents’ oral microbiome is a determinant of the subgingival microbial colonization of their children, and parental periodontal disease is associated with the acquisition of a pathogen-rich microbiome in their offspring. Furthermore, these dysbiotic microbiota acquired by children of periodontitis patients at an early age are resilient to shift and the community structure is maintained even after controlling the hygiene status.

## Materials and method

This study was conducted at Piracicaba Dental School, University of Campinas, Brazil, and was approved by the Ethical Committee in Research of the Piracicaba Dental School (process 079/2013). The study protocol was registered at clinicaltrial.gov under the ID NCT03933514, was developed following the STROBE guidelines for observational studies, and written informed consent and assent obtained from parents and children before enrollment. The study was designed as an age- and gender-matched case–control interventional study.

Eighteen children whose parents were diagnosed with GAgP (periodontitis children) and eighteen children with both parents periodontally healthy (healthy children) were recruited. This investigation used the inclusion criteria described in previous studies^15,16^:

1. *Systemically healthy* children between 6 to 12 years of age, in whom the permanent first molars and central incisors fully erupted.
2. Periodontitis group: Parents classified as GAgP at the moment of diagnosis (currently classified as grade C periodontitis^2^). Parents who were < 35 years of age at the time of diagnosis; at least 8 teeth with probing depth (PD) and clinical attachment level (CAL) > 5 mm (with at least 2 sites with PD > 7 mm) at diagnosis; at least 20 teeth in the oral cavity; good systemic health.
3. Health group: Parents with no history of attachment loss. Absence of periodontal pockets: gingival sulcus with PD < 4 mm; absence of radiographic proximal bone loss; at least 20 teeth in the oral cavity; good systemic health.

The exclusion criteria for children were: (1) use of orthodontic appliance; (2) the use of antibiotics and anti-inflammatory medication six months before the study; (3) alteration in the motor condition that modifies brushing habits; (4) history of current or past smoking in children or parents.

First, parents with periodontitis and their children were selected. After the selection of the periodontitis group, the healthy group was chosen to frequency match the age and gender of the periodontitis group. The children for the healthy group were paired with the children from the periodontitis groups, and the families were selected. Only one of the parents of a healthy group was selected per family, and the choice of father or mother was made based on the age and gender of the adults to pair the healthy with the periodontitis group.

At baseline, children and parents were clinically evaluated, and samples of subgingival biofilm were collected. After that, children were included in a plaque control program for three months. The protocol consisted of professional plaque control at the initial visit and oral hygiene instructions that included brushing with Bass technique three times per day and flossing. One month later, compliance with home care was monitored, and oral hygiene habits reinforced as needed. Three months after the beginning of plaque control, all children were reassessed for their clinical condition, and samples were collected. All children completed the plaque control.

The clinical periodontal metrics included plaque index—PI^37^, gingival index—GI^37^, probing depth—PD, bleeding on probing—BoP^38^. The examination was performed by an experienced and calibrated examiner (MFM—Intra-class correlation = 92% of PPD).

### Clinical data analysis

For gender distribution and age, Chi-square and Student's t-tests were used. Because of the non-normal distribution of clinical data (detected with the Shapiro–Wilk test), the Mann–Whitney test was used in the group's comparison for parents and children, and Wilcoxon test was used to compare baseline and three months in children. All tests considered alpha = 5%.

### Microbiome analysis

Subgingival biofilm was collected from 2 incisors and 2 first molars of children and parents by the same examiner (MFM) that carried out the clinical evaluation. The collected sites were the ones with the deepest PD of one molar and one incisor in the maxillary arch and one molar and one incisor in the mandibular. Following supragingival biofilm removal and isolation with cotton rolls, a sterile paper point (Nº35) was inserted into the bottom of the periodontal pocket/gingival sulci for 30 s. The paper points were placed into sterile tubes containing 300 μL of Tris-EDTA 0.5 mM and stored at − 20 °C until laboratory evaluation.

The plaque was removed from the paper points by adding 200 ml of phosphate-buffered saline (PBS) and vortexing for 1 min. The paper points were removed, and DNA isolated using a Qiagen MiniAmp kit (Valencia, CA) according to the manufacturer's instructions, after a 90-min incubation with lysozyme (2 mg/ml) (Thermo Fisher Scientific). Two regions of the 16S rRNA genes were sequenced: V1–V3 (27F: 5′-GAAKRGTTYGATYNTGGCTCAG and 519R: 5′-ACGTNTBACCGCDGCTGCTG) and V4–V5 (515F: 5′-GAGTGCCAGCMGCCGCGGTAA and 806R: 5′-ACGGACTACHVGGGTWTCTAAT). The 16S rRNA amplicons were quantified using the Quant-iT PicoGreen dsDNA reagent and kit (Invitrogen). Equimolar concentrations of each amplicon were pooled and sequenced on the 2 × 250 Miseq run (Illumina). The sequenced data were deposited in the Sequence Read Archive (SRA) database (accession number: PRJNA606501). Negative and positive controls (defined culture mixture) were used in all runs. Two primers were used since each primer can detect a genera range that the other fails to recover. Together they allow the recovery of a wider microbiome range than is possible with a single primer alone. However, some genera are picked up by both primers. Thus, to prevent overcounting, the number of sequences assigned to an OTU by both primers was reduced by half. Primer averaging was carried out as previously described^39^ using the implementation in the PhyloToAST software suite^40^. Analyses were conducted using QIIME1.9.0^41^ and PhyloToAST. The sequences were binned by sample, aggregated, and de novo operational taxonomic units (OTUs) were identified. In order to be retained in the dataset, the sequence had to be detected at least once in at least 5% of the samples. Sequences were clustered into distinct OTUs at 97% similarity using the UCLUST method^42^. Chimeric sequences were depleted using ChimeraSlayer (v. 1.9.0, identify_chimeric_seqs.py). Sequences with an average quality score of 30 over a sliding window of 50 bp and length > 200 bp were assigned a taxonomic identity by alignment to the HOMD database as of 02/02/2018^43^ using the Blastn algorithm at 97% identity. Alpha (within-group) and beta (between-group) diversity were computed. Since emergent evidence does not support rarefying the microbiome to compensate for sequencing effort^44^, we used cumulative sum scaling (CSS) normalization from the Bioconductor package metagenomeSeq. Chao1 and Shannon indexes were used as an estimator of alpha diversity and significance of group-wise clustering interrogated using the Mann–Whitney test. The core species were characterized using Qiime's script (core_microbiome.py) when species were present in at least 75% of the patients in each group. The Interactive Tree Of Life (IToL) webserver was used for the graphic visualization of the phylogenetic tree^45^, and information of relative abundance (using bars outside the tree) and differential abundance (marking the species with a statistically significant difference between groups with colored names) of species were included in the tree. Bacterial network correlations were determined by significant pairwise using the SparCC pipeline (*p* < 0.01, r > 0.75)^46^, and network graphs were visualized in Gephi^47^. Beta diversity was estimated using the Morisita-Horn dissimilarity distance matrices, the Linear Discriminant Analysis (LDA) was performed on distance matrices, and the clustering was interrogated using Adonis with 999 permutations. LDA plot was generated using the R package ggplot. The Bioconductor package for R, DESeq2, was used to perform differential abundance analysis of the annotated taxa^48^. This function uses a negative binomial distribution of raw counts to estimate between-group differences while accounting for sampling effort (library size) and dispersion of each category (taxon or functional gene). *P*-values were adjusted for multiple testing (FDR < 0.1, FDR-adjusted Wald Test). SourceTracker 0.9.5 software in QIIME was utilized to predict the source of a child's microbial community.

## Supplementary Information

Supplementary Information 1.

Supplementary Information 2.

## Data Availability

The datasets generated and analyzed during the current study are available in the Sequence Read Archive (SRA) repository, https://www.ncbi.nlm.nih.gov/sra/PRJNA606501.
